# Optimization of Composite Enzymatic Extraction, Structural Characterization and Biological Activity of Soluble Dietary Fiber from *Akebia trifoliata* Peel

**DOI:** 10.3390/molecules29092085

**Published:** 2024-05-01

**Authors:** Ya Song, Guoshun Sun, Dian Wang, Jin Chen, Jun Lv, Sixia Jiang, Guoqiang Zhang, Shirui Yu, Huayan Zheng

**Affiliations:** 1 Department of Food Science and Engineering, Moutai Institute, Renhuai 564507, China; songya_1990@163.com (Y.S.); sunguoshun@email.ncu.edu.cn (G.S.); wd020606@163.com (D.W.); chenj200121@163.com (J.C.); lyuj123@yeah.net (J.L.); 18585206504@163.com (S.J.); zhangguoqiang@mtxy.edu.cn (G.Z.); ysr312004@126.com (S.Y.); 2 State Key Laboratory of Food Science and Resources, Nanchang University, Nanchang 330047, China; 3 Engineering Technology Research Center of Health Wine Brewing, Renhuai 564507, China; 4 Talent Cultivation Center of Moutai Institute on Characteristic Food Resource Utilization, Renhuai 564507, China

**Keywords:** *Akebia trifoliata*, soluble dietary fiber, extraction, structure, antioxidant activity, functional properties

## Abstract

In order to reduce the waste of *Akebia trifoliata* peel and maximize its utilization, in this study, on the basis of a single-factor experiment and the response surface method, the optimum technological conditions for the extraction of soluble dietary fiber from *Akebia trifoliata* peel with the compound enzyme method were obtained. The chemical composition, physical and chemical properties, structural characterization and biological activity of the purified soluble dietary fiber (AP-SDF) from the *Akebia trifoliata* peel were analyzed. We discovered that that the optimum yield was 20.87% under the conditions of cellulase addition 600 U/g, enzymolysis time 100 min, solid–liquid ratio 1:24 g/mL and enzymolysis temperature 51 °C. At the same time, AP-SDF was a porous network structure cellulose type I acidic polysaccharose mainly composed of arabinoxylan (36.03%), galacturonic acid (27.40%) and glucose (19.00%), which possessed the structural characteristic peaks of the infrared spectra of polysaccharides and the average molecular weight (Mw) was 95.52 kDa with good uniformity. In addition, the AP-SDF exhibited high oil-holding capacity (15.11 *g*/*g*), good water-holding capacity and swelling capacity, a certain antioxidant capacity in vitro, hypoglycemic activity in vitro for α-glucosidase inhibition and hypolipidemic activity in vitro for the binding ability of bile acids and cholesterol. These results will provide a theoretical basis for the development of functional products with antioxidant, hypoglycemic and hypolipidemic effects, which have certain application value in related industries.

## 1. Introduction

The vine *Akebia trifoliata*, a member of the *Lardizabalaceae* family, is widely distributed across Asia, including China, Korea and Japan [1]. Its fruit, known as “Bayuezha” in China, has been recognized as a significant medicinal herb for 2000 years [2]. The fruit is rich in protein, organic acids, vitamins, minerals, flavonoids and other active substances, contributing to its medicinal properties such as anti-aging, immunity enhancement, anti-tumor, blood pressure reduction, anti-bacterial, anti-inflammatory, and diuretic effects [3]. Recently, the demand for *Akebia trifoliata*, both as an herbal medicine and a fruit, has increased dramatically [4]. It has been utilized in the production of fruit vinegar, fruit oil, juice, and has potential applications in pharmaceuticals and cosmetics [5]. The peel of *Akebia trifoliata*, which constitutes about 60% of the fruit’s total weight, is often discarded during daily consumption and processing, resulting in a significant underutilized by-product [6]. Previous studies have reported that *Akebia trifoliata* peel is rich in sugars, pectin, and polyphenols [5], highlighting the need for methods to transform this waste into high-value products, thereby promoting resource efficiency.

Dietary fiber (DF), an indigestible carbohydrate polymer with three or more monomeric units found in plants, can be classified into soluble dietary fiber (SDF) and insoluble dietary fiber (IDF) based on solubility [7]. SDF, a plant polysaccharide that includes pectin, gum, dextran, and guar gum [8], has attracted more and more attention due to its exceptional functional properties. Research has shown that S SDF not only possesses antioxidant properties and aids in reducing blood sugar, blood lipids and blood pressure [9] but also helps prevent heart and cardiovascular diseases [10], regulate gut microbiota, and promote probiotic growth [11]. Currently, the main extraction methods for SDF include hot water, chemical, physical, enzymatic, and microbial fermentation, each with distinct characteristics [8,12]. The enzymatic method is preferred due to its high extraction efficiency, mild conditions, minimal environmental pollution, and absence of chemical residue [13].

To date, most studies have primarily assessed the biological activity and functional role of *Akebia trifoliata*. Jiang et al. [6] developed a novel active packaging film from the peel extract of *Akebia trifoliata*, demonstrating high UV light resistance, antibacterial activity, and enhanced mechanical stability. Luo et al. [14] reported that the peel extract of *Akebia trifoliata* has a certain antioxidant capacity and can be used to prevent some diseases caused by oxidative stress. Jiang et al. [15] found that dietary fiber extracted from *Akebia trifoliata* seeds exhibited different functional properties depending on the extraction method, with ultrasonication-assisted enzymatic hydrolysis most effectively enhancing the overall performance of dietary fiber. However, up to now, study of the extraction, structural characterization and biological activity of soluble dietary fiber from *Akebia trifoliata* peel has not been reported.

Therefore, this study aims to determine the optimal conditions for extracting SDF from *Akebia trifoliata* peel with the compound enzyme method, combining single-factor experiments with response surface methodology. We also investigated the chemical composition and structural characteristics of the obtained AP-SDF and evaluated its functional properties and in vitro antioxidant, hypoglycemic and hypolipidemic activities. This research may provide a theoretical foundation for further exploration and application of SDF from *Akebia trifoliata* peel.

## 2. Results and Discussion

### 2.1. Single-Factor Experimental Results

#### 2.1.1. Effect of Enzyme Addition on SDF Yield

**Figure 1:**
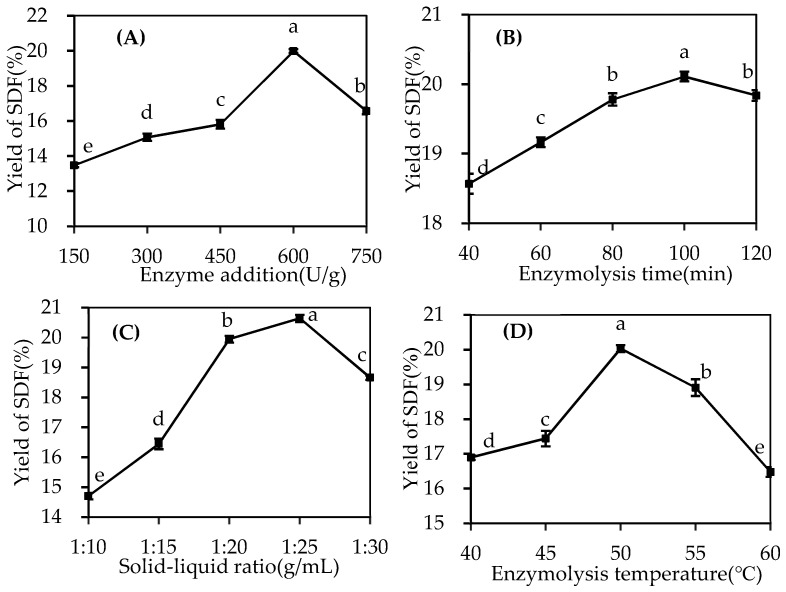
Effects of enzyme addition (**A**), enzymolysis time (**B**), solid–liquid ratio (**C**) and enzymolysis temperature (**D**) on SDF yield. Different letters in the same figure indicate significant differences (*p* < 0.05).

As illustrated in Figure 1A, the amount of cellulase added significantly affects the yield of SDF (*p* < 0.05). The yield of SDF initially increases with the addition of the enzyme, reaching a maximum value of 19.99% at an enzyme addition of 600 U/g, after which it decreases. Cellulase enhances the yield by destroying the peel’s cell wall and hydrolyzing IDF. However, excessive enzyme addition reduces its degree of polymerization, preventing precipitation by alcohol [10]. Cheng et al. [16] found a similar effect when using cellulase to extract polysaccharides from *Schisandra chinensis* Baill. Therefore, selecting an appropriate amount of enzyme to maintain the hydrolysis balance is crucial for yield improvement. Consequently, 450–750 U/g were selected for subsequent response surface experiments.

#### 2.1.2. Effect of Enzymolysis Time on SDF Yield

The enzymolysis time significantly impacts the SDF yield (*p* < 0.05), as shown in Figure 1B. The yield of SDF continuously increases with the extension of enzymolysis time, reaching a peak value of 20.11% at 100 min. However, extending the enzymolysis time further results in a yield decline. This can be attributed to insufficient enzymolysis due to a short enzymolysis time and a partial hydrolysis of the enzyme and SDF due to a longer time [17]. Lin et al. also observed similar results [18]. Therefore, considering an appropriate enzymolysis time is crucial to prevent the hydrolysis of SDF. Hence, 80–120 min was selected for further optimization experiments.

#### 2.1.3. Effect of Solid–Liquid Ratio on SDF Yield

As evident in Figure 1C, the solid–liquid ratio also take a significant effect on the SDF yield (*p* < 0.05). The yield increases with the solid–liquid ratio from 1:10 to 1:25, reaching a maximum of 20.64% at 1:25 g/mL. This increase is due to the enhanced concentration difference between inside and outside the cell’s interior and exterior, improving dissolution efficiency [19]. However, the SDF yield decreases when the liquid–solid ratio is further increased, as an excessive solid–liquid ratio reduces the polysaccharide concentration and the cellulase reaction efficiency, weakening the destructive enzyme’s effect on plant cell walls and reducing the SDF yield. Thus, 1:20–1:30 g/mL were chosen for further study.

#### 2.1.4. Effect of Enzymolysis Temperature on SDF Yield

The enzymolysis temperature is another experimental factor that significantly affects the SDF yield (*p* < 0.05), as shown in Figure 1D. The yield of SDF increases over the enzymatic hydrolysis temperature range of 40–50 °C but decreases sharply over the temperature range of 50–60 °C. The maximum yield (20.03%) of SDF was observed at 50 °C. Relevant reports have pointed out that increasing the temperature appropriately can enhance the activity of the enzyme and promote mass transfer. However, excessive temperature inactivates the enzyme, leading to the thermal degradation of polysaccharides, increased solution viscosity and, finally, a reduced extraction rate [20]. Considering these factors, 45–55 °C was adopted for subsequent response surface experiments. 

### 2.2. Response Surface Analysis Results

#### 2.2.1. Model Fitting and Variance Analysis

Following 29 runs of RSM optimization experiments, the relationship between SDF yield and experimental factors is presented in Table 1. The second-order polynomial equation of SDF yield (*Y*) with enzyme addition (*X*_1_), enzymolysis time (*X*_2_), solid–liquid ratio (*X*_3_) and enzymolysis temperature (*X*_4_) is as follows:

*Y* = 20.60 + 0.20*X*_1_ − 8.333E − 004*X*_2_ − 0.70*X*_3_ + 0.55*X*_4_ + 0.35*X*_1_*X*_2_ − 0.057*X*_1_*X*_3_ − 0.035*X*_1_*X*_4_ + 0.055*X*_2_*X*_3_ + 0.030*X*_2_*X*_4_ + 0.34*X*_3_*X*_4_ − 3.72*X*_1_^2^ − 0.23*X*_2_^2^ − 1.11*X*_3_^2^ − 1.67*X*_4_^2^ (1)

The ANOVA results for the quadratic multiple regression model are shown in Table 2. The regression model was highly significant (*F* = 121.05, *p* < 0.0001), while the lack of fit (*p* = 0.97 > 0.05) was insignificant relative to the pure error, indicating a good fit of the regression equation to the experiment. The determination coefficient (*R*^2^) of the model was 0.9918, and the adjusted determination coefficient (*R*^2^_Adj_) was 0.9836, suggesting that this model could explain 98.36% of the response value changes. The coefficient of variation (C.V.%) was 1.43%, and the Adeq Precision was 33.616, indicating the model’s suitability for theoretical prediction [21]. According to the *p*-value of each factor, the linear coefficients *X*_1_, *X*_3_ and *X*_4_, the interaction coefficients *X*_1_*X*_2_ and *X*_3_*X*_4_, and the quadratic coefficients *X*_1_^2^, *X*_2_^2^, *X*_3_^2^ and *X*_4_^2^ all had significant effects (*p* < 0.05). In the model, the negative quadratic coefficient indicates a downward parabolic opening of the model with a maximum value. The effects of various factors on the yield of SDF were in the order of solid–liquid ratio (*X*_3_) > enzymolysis temperature (*X*_4_) > enzyme addition (*X*_1_) > enzymolysis time (*X*_2_).

#### 2.2.2. Response Surface Interaction Analysis

**Figure 2:**
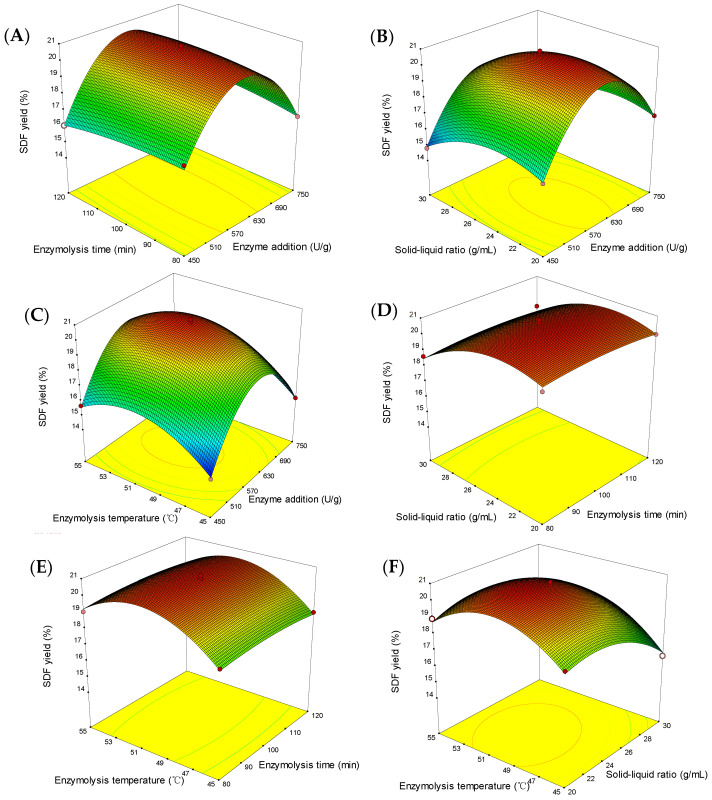
Response surface diagram. The interaction of enzyme addition and enzymolysis time (**A**), enzyme addition and solid–liquid ratio (**B**), enzyme addition and enzymolysis temperature (**C**), enzymolysis time and solid–liquid ratio (**D**), enzymolysis time and enzymolysis temperature (**E**) and solid–liquid ratio and enzymolysis temperature (**F**) and effects on SDF yield.

The response surface reflects the effect of the interaction between the two variables on the SDF yield, keeping the others constant at the central levels [22]. As seen in Figure 2, the response value first increased and then decreased with each factor level increasing. The contour plots in Figure 2A,F were elliptical, indicating significant interactions (*p* < 0.05) between enzyme addition and enzymolysis time *(X*_1_*X*_2_) and solid–liquid ratio and enzymolysis temperature (*X*_3_*X*_4_). The slope of enzyme addition (*X*_1_) was steeper than that of enzymolysis time (*X*_2_), and the contour plot was denser, indicating a more significant effect on the response value (*Y*). Similarly, the effect of the solid–liquid ratio (*X*_3_) on the response value (*Y*) was more significant than that of the enzymolysis temperature (*X*_4_). These results were consistent with the ANOVA results. 

#### 2.2.3. Optimization and Verification of Extraction Conditions

The obtained second-order polynomial equation was calculated and analyzed using Design Expert v10 software. Considering the feasibility of practical operation, the optimal conditions were determined as follows: cellulase addition amount 600 U/g, enzymolysis time 100 min, solid–liquid ratio 1:24 g/mL, enzymolysis temperature 51 °C. Under these conditions, six parallel experiments were conducted to verify the optimized results. The average yield of SDF was 20.87%, which was close to the theoretical value (20.74%), indicating a good fit and demonstrating that the model was accurate and reliable for optimizing the SDF extraction process.

### 2.3. Characterization of AP-SDF

#### 2.3.1. Chemical Composition, Monosaccharide Composition and Mw of AP-SDF

The chemical composition of AP-SDF is shown in Table 3. The purified AP-SDF contained a high uronic acid content (34.90%) and a total sugar content of 52.46%. Despite the removal of soluble proteins by the Sevag method in this study, a low quantity of proteins (8.33%) was also found, indicating that AP-SDF was an acidic polysaccharide and the existence of minor polysaccharide–protein complexes, which often exhibit favorable bioactivities [23].

**Figure 3:**
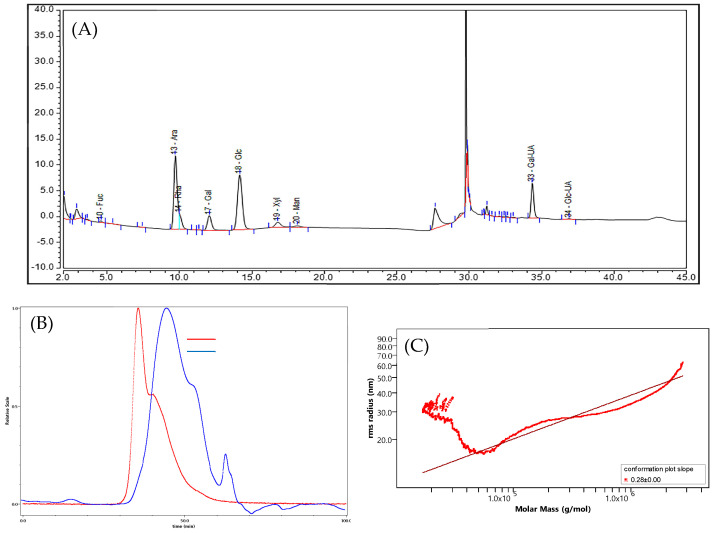
Chromatogram of AP-SDF (**A**), HPSEC chromatograms of AP-SDF (**B**) and molecular configuration diagram of AP-SDF (**C**). LS represents multi-angle laser light scattering detector, and RI represents difference detector.

As shown in Table 3 and Figure 3A, AP-SDF is mainly composed of arabinose (36.03%), galacturonic acid (27.40%) and glucose (19.00%), with fucose, rhamnose, galactose, xylose, mannose and glucuronic acid as minor components. This finding is consistent with the ATKP (mainly composed of glucose, arabinose and galactose) reported by Li et al. [24] and the results for pectin from *Akebia trifoliata* var. *australis* peel (high galacturonic acid) of Jiang et al. [5]. The results suggested that AP-SDF was an acidic polysaccharide, consistent with the conclusion of the chemical composition analysis. Based on previous studies, the physiological activity of polysaccharides may vary depending on the type and ratio of the monosaccharides. Polysaccharides with high levels of galacturonic acid may have potent scavenging ability [25], suggesting that AP-SDF might have good bioactivity. 

The elution curves of AP-SDF under multi-angle laser light scattering (LS) detector and refractive index (RI) detector are shown in Figure 3B. A large broad peak was detected by both LS and RI, with two small peaks under the detection of RI, while LS has a small peak. This was due to the fact that low concentrations of polysaccharide fragments could only be detected by RI [26]. These indicated the existence of one major constituent and lower molecular weight polysaccharide fragments. Figure 3C demonstrates the molecular conformation diagram of AP-SDF, which is expressed by molar mass and root mean square radius. The slope of AP-SDF was calculated to be 0.28, as shown in the molecular conformation diagram, implying that AP-SDF showed a globular conformation [26]. As seen from Table 3, the molecular weight of AP-SDF (95.52 kDa) is larger than the pectin from the *Akebia trifoliata* var. *australis* fruit peel reported by Cai et al. [27]. The reason is that citric acid has a stronger degradative effect on molecular weight relative to enzymatic conditions [5]. It has been shown that polysaccharides with lower molecular weight exhibit higher biological activity [25]. This suggests the possibility that the relatively lower molecular weight AP-SDF may possess strong biological activity. In addition, Table 3 shows that AP-SDF has a relatively low polydispersity coefficient (2.57), indicating that it has a relatively concentrated and uniform molecular weight distribution [8].

#### 2.3.2. FT-IR Spectrum of AP-SDF

**Figure 4:**
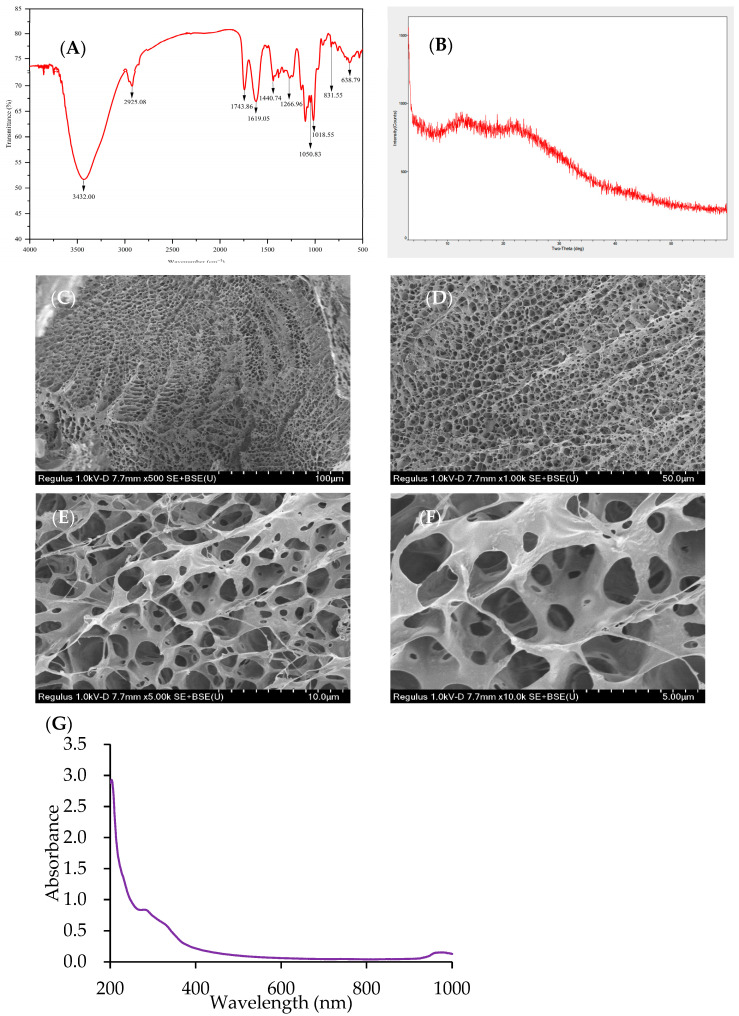
FT-IR spectrum (**A**), XRD diagrams (**B**), SEM images × 500 (**C**), SEM images × 1000 (**D**), SEM images × 5000 (**E**), SEM images × 10,000 (**F**) and UV–vis spectrum (**G**) of AP-SDF.

The FT-IR spectrum of AP-SDF is presented in Figure 4A. AP-SDF presents a wide and smooth absorption peak at about 3432.00 cm^−1^ caused by the stretching vibration of the -OH, indicating the presence of more hydroxyl groups and bound water molecules. The peak at 2925.08 cm^−1^ is attributed to the C-H stretching vibration of methyl and methylene [8]. These two absorption peaks belong to characteristic groups of polysaccharides. The absorption peaks at 1743.86 cm^−1^ and 1619.05 cm^−1^ caused by C=O stretching vibration of ester carbonyl groups and ionic carboxyl groups, respectively, represent the existence of uronic acid [27], consistent with the results of the analysis of chemical composition and monosaccharide composition (Table 3). The characteristic peaks at 1440.74~1266.96 cm^−1^ illustrate the bending vibration of C–H of carbonyl groups [22,28]. In addition, the wavelength range from 900 to 1200 cm^−1^ is considered to be the fingerprint area of carbohydrates. The absorption peaks at 1050.83 cm^−1^ and 1018.55 cm^−1^ are attributed to the C-O-H and C-O-C stretching vibrations of the pyranose ring [10]. The weak peaks at 831.55 cm^−1^ and 638.79 cm^−1^ indicate that the configuration of AP-SDF is dominated by α-glycosidic bonds [23]. 

#### 2.3.3. XRD of AP-SDF

As shown in Figure 4B, the crystalline structure of AP-SDF was determined by X-ray diffraction. AP-SDF showed broad diffraction peaks at 13.23 ° and 22.16 °. This indicated that AP-SDF exhibited an amorphous structure and presented typical type cellulose I crystals. The results were consistent with Jiang et al. [5].

#### 2.3.4. SEM of AP-SDF

The surface microstructure of AP-SDF was explored by SEM at different magnifications from 500× to 10,000× (Figure 4C–F). AP-SDF showed a regular honeycomb-type network structure at the magnification of 500× and 1000× while presenting an interconnected loose and porous network structure at a magnification of 5000× and 10,000×, which may be due to enzymatic hydrolysis extending the pores in the plant cell walls [25]. The observed microstructure was consistent with the CEP-Ag results of Yu et al. [29]. In addition, the porous honeycomb structure of AP-SDF would influence the physicochemical characteristics and functional properties. 

#### 2.3.5. UV–Visible Spectroscopy of AP-SDF

The UV spectra of AP-SDF (Figure 4G) exhibited minimal absorbance within the 240–400 nm range, indicating the potential presence of glycoside–protein or glycoside–nucleic acid complexes in AP-SDF [26]. This finding aligns with the chemical composition results (Table 3). 

### 2.4. Functional Properties of AP-SDF

The physicochemical properties of SDF can vary based on the source and preparation method [15]. As shown in Table 4, the WHC and SC of AP-SDF were 1.31 ± 0.16 *g*/*g* and 4.68 ± 0.47 mL/g, respectively. These values were lower than those of soluble dietary fibers from *Tremella fuciformis* (9.26 ± 0.20 *g*/*g* and 10.92 ± 0.17 mL/g) [9] and dietary fiber from maca liquor residue (16.29 ± 0.69 *g*/*g* and 26.17 ± 0.29 mL/g) [30]. The reduced WHC and SC could be attributed to the enzymatic hydrolysis-induced destruction of the matrix structure and polysaccharide linkages. The OHC of AP-SDF was 15.11 ± 0.60 *g*/*g*, significantly higher than that of SDF from soybean residue (2.47 ± 0.34 *g*/*g*) [21] and coffee peel (2.66 ± 0.08) [31]. This could be attributed to its loose and porous network structure (Figure 4C–F). Dietary fiber can reduce serum cholesterol levels by absorbing oil in the intestine [9]. The relatively high OHC suggests that AP-SDF may play a crucial role in lowering blood lipids.

### 2.5. Bioactivity of AP-SDF

#### 2.5.1. Antioxidant Activities of AP-SDF

**Figure 5:**
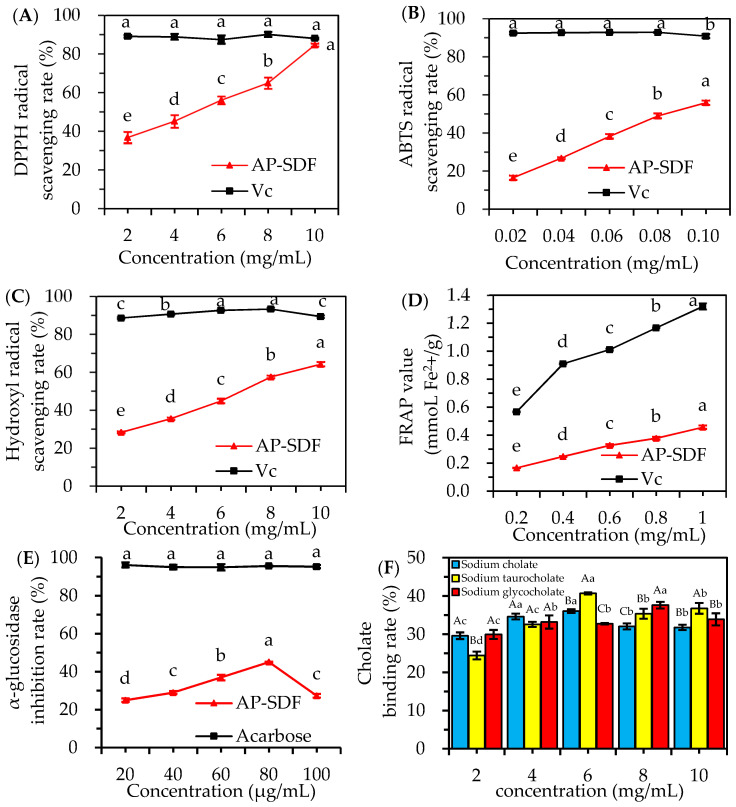
Bioactivity of AP-SDF. DPPH radical scavenging activity (**A**), ABTS radical scavenging activity (**B**), hydroxyl radical scavenging activity (**C**), FRAP value (**D**), α-glucosidase inhibitory activity (**E**) and cholate binding activity (**F**). Different lowercase letters with the same sample at different concentrations and different uppercase letters with different samples at the same concentration indicate significant differences (*p* < 0.05).

Figure 5 shows a positive correlation between the in vitro antioxidant capacity of AP-SDF and its concentration. However, all four in vitro antioxidant indexes of AP-SDF were lower than those of Vc. DPPH and ABTS are commonly used to evaluate the in vitro antioxidant activity of natural compounds. Free radicals are closely associated with the peroxidation process in the human body. A sample’s ability to scavenge free radicals is indicative of its antioxidant activity [26]. Figure 5A shows the DPPH radical scavenging ability of AP-SDF. At a concentration of 10 mg/mL, the radical scavenging rates reached 84.67%, which was close to that of VC (88.07%). As shown in Figure 5B, the ABTS free radical scavenging capacity exhibited concentration-dependence, with the scavenging rate increasing from 16.53 % to 55.92% in the 0.02–0.1 mg/mL range. Hydroxyl radical, an extremely active oxygen free radical, is one of the main factors causing oxidative damage to the body [25]. The hydroxyl radical scavenging capacities of AP-SDF are presented in Figure 5C. The scavenging rate gradually increased as the concentration of AP-SDF increased in the test range, reaching 64.28% at a concentration of 10 mg/mL. Our results showed that the FRAP value of AP-SDF exhibited dose-dependence (Figure 5D). The FRAP value of AP-SDF reached 0.46 mmoL Fe^2+^/g at a concentration of 1 mg/mL. As a positive control, Vc demonstrated a higher reducing capacity (1.32 mmoL Fe^2+^/g).

The IC_50_ values of AP-SDF for ABTS free radicals, DPPH free radicals and hydroxyl free radicals were 0.08 mg/mL, 4.00 mg/mL and 6.28 mg/mL, respectively. The highest ABTS radical scavenging effect was observed for AP-SDF, followed closely by DPPH and hydroxyl radicals. This may be due to the different mechanisms of AP-SDF’s effect on the scavenging of different free radicals, resulting in variable scavenging capacity. The antioxidant activity of polysaccharides is influenced by their monosaccharide composition, structural characteristics, and Mw [8]. Feng et al. [22] found that rhamnose and mannose content were positively correlated with DPPH and hydroxyl free radical scavenging capacity, whereas polysaccharides enriched in galactose and arabinose had a stronger ABTS free radical scavenging capacity. Meng et al. [32] found that glucose content was negatively correlated with the hydroxyl radical scavenging ability. Furthermore, it is generally believed that polysaccharides with high galacturonic acid content and lower molecular weight have considerable antioxidant activity, owing to the ability of galacturonic acid to bind metal ions and enhance antioxidant effects by providing hydrogen and electron transfer [22], while low Mw polysaccharides reveal more reducing ends to terminate the free radical reaction [33]. Notably, these findings are consistent with our results (Table 3 and Figure 5A–D), indicating that AP-SDF has excellent antioxidant activity.

#### 2.5.2. α-Glucosidase Inhibitory Activity of AP-SDF

α-Glucosidase, an enzyme that degrades carbohydrates into monosaccharides, thereby increasing blood glucose levels, is a potential target for type 2 diabetes treatment. The inhibitory effect of AP-SDF on α-glucosidase is depicted in Figure 5E. Within the concentration range of 20–100 μg/mL, AP-SDF demonstrated an inhibitory activity that first increased and then decreased, peaking at 80 μg/mL (45.06%). This was lower than the 95.59% inhibition exhibited by the positive control acarbose. This result suggests that AP-SDF possesses a certain degree of α-glucosidase inhibitory activity. Previous studies have found that polysaccharides with a high arabinose content exhibit significant α-glucosidase inhibitory activity [34], which aligns with the monosaccharide composition of AP-SDF (36.03% of arabinose) (Table 3). Currently, acarbose is used to treat diabetes, but it has toxicity and several side effects [35]. Therefore, AP-SDF, a natural and non-toxic extract with α-glucosidase inhibitory ability, could potentially serve as a source of hypoglycemic functional foods or drugs.

#### 2.5.3. Hypolipidemic Activity of AP-SDF

Dietary fiber can exert hypolipidemic effects by adsorbing bile acids and cholesterol, thereby promoting the elimination of bile acids and cholesterol degradation in the small intestine [36]. As shown in Figure 5F, the binding capacity of AP-SDF for three sodium cholates first increased with concentration and then plateaued. Sodium cholate and sodium taurocholate reached their maximum binding capacity at 6 mg/mL (36.06% and 40.69%, respectively), while sodium glycocholate peaked at 8 mg/mL (37.60%). The bile acid-binding capacity of AP-SDF may be attributed to its lower molecular weight and higher galacturonic acid content (Table 3) [37], suggesting that AP-SDF can promote the conversion of cholesterol to bile acids and play a crucial role in hypolipidemic processes.

Experiments simulating gastric (pH = 2) and small intestinal (pH = 7) environments in vivo revealed the cholesterol adsorption effect of AP-SDF. The adsorption at pH 7 (156.41 ± 7.52 mg/g) was higher than at pH 2 (125.34 ± 4.20 mg/g), consistent with the results of *Cerasus humilis* pectin [38]. Under acidic conditions, a large number of hydrogen ions repel the positive charges of SDF and cholesterol. Since AP-SDF contains galacturonic acid, the carboxyl group does not dissociate under acidic conditions. However, when the pH increases, the carboxyl group dissociates and transforms into a carboxyl anion (RCOO^−^), which has a stronger binding capacity to cholesterol, thereby enhancing the cholesterol adsorption capacity of AP-SDF [8]. After enzyme modification, AP-SDF exhibits an interconnected porous honeycomb network structure (Figure 4C–F) after enzyme modification, thereby increasing its capacity to absorb more cholesterol. Furthermore, the exposure of hydroxyl groups leads to an increase in accessible hydrogen bonding, which in turn enhances the binding capacity of AP-SDF. As a result, AP-SDF may be used to reduce cholesterol absorption and lower blood lipid levels.

## 3. Materials and Methods

### 3.1. Materials and Reagents

*Akebia trifoliata* fruits were sourced from a local plantation in Zunyi city, Guizhou, China. Cellulase (15,000 μ/g), α-amylase (40,000 μ/g), papain (800,000 μ/g), α-glucosidase, pepsin, pancreatic lipase, 1,1-diphenyl-2-picrylhydrazine (DPPH), 2,2′-azinobis (3-ethylbenzothiazoline-6-sulfonic acid) (ABTS), 2,4,6-tripyridyltriazine (TPTZ), p-nitrophenyl-α-D-glucopyranoside (PNPG) and cholesterol were purchased from Shanghai Yuanye Bio-Technology Co., Ltd., Shanghai, China. Monosaccharide standards were obtained from Sigma Chemical Co., St. Louis, MO, USA. All other chemicals and reagents of analytical grade were obtained from Sinopharm Chemical Reagent, Shanghai, China.

### 3.2. Material Preparation

Prior to the extraction, the peel of fresh and mature *Akebia trifoliate* was washed, naturally dried, and then oven-dried at 50 °C until a constant weight was achieved. The dried peel was crushed and sieved through a 60-mesh sieve. As previously described [39], the peel powder was soaked in boiling 95% ethanol for 20 min, washed with 80% ethanol to remove low molecular sugars and organic acids and to inactivate enzymes. The residue was dried at 50 °C, crushed, sieved through a 60-mesh screen, and oven-dried at 50 °C to a constant weight.

### 3.3. Extraction of SDF from Akebia trifoliata Peel

The compound enzymatic extraction was performed based on a previous report [13], with minor modifications. Briefly, the powder of *Akebia trifoliata* peel was thoroughly mixed with distilled water (1:20, *w*/*v*). The pH was adjusted to 7, 600 U/g papain was added, and the mixture was shaken well to ensure thorough mixing. It was then water-bathed at 70 °C for 30 min, and the enzyme was inactivated at 100 °C for 10 min. After cooling, the pH was adjusted to 6.5, 30 U/g α-amylase was added, and the mixture was water-bathed at 85 °C for 30 min. After inactivation and cooling, the pH was adjusted, a certain amount of cellulase was added, and timed enzymatic hydrolysis was performed in a constant-temperature water bath. The enzyme was inactivated at approximately 100 °C for 10 min after enzymatic hydrolysis. The resulting solution is the peel’s enzymatic hydrolysate. The enzymatic hydrolysate was then centrifuged (Scientz Biotechnology Inc., Ningbo, China) at 1776× *g* for 10 min and subsequently centrifuged twice with 70 °C hot water. The combined supernatant was collected and concentrated to a certain volume. Subsequently, four times the volume of 95% ethanol was used for precipitation at 4 °C for 24 h, centrifuged (2495× *g*, 10 min), and washed twice sequentially with 80% and 95% ethanol. Finally, the precipitate was lyophilized until completely dried to obtain crude SDF. The yield of crude SDF was calculated as follows:

(2) $$Yield\;(\%)=\frac{m}{M}\times 100$$

where *m* is the weight of crude SDF (g), and *M* is the weight of the powder of the *Akebia trifoliata* peel (g).

### 3.4. Optimization of Extraction Condition

#### 3.4.1. Single-Factor Experiment

Refer to 3.3 for the extraction method. One gram of *Akebia trifoliata* peel powder was weighed. The fixed process parameters were as follows: the addition amount of cellulase was 600 U/g, the enzymolysis time was 100 min, the enzymolysis temperature was 50 °C, the enzymolysis pH was 5, and the solid–liquid ratio was 1:20 g/mL. The addition of cellulase (150, 300, 450, 600, 750 U/g), enzymolysis time (40, 60, 80, 100, 120 min), solid–liquid ratio (1:10, 1:15, 1:20, 1:25, 1:30 g/mL) and enzymolysis temperature (40, 45, 50, 55, 60 °C) were investigated separately to evaluate the influence of these factors on SDF yield.

#### 3.4.2. Response Surface Methodology (RSM) Design

The RSM method was used to investigate the relationship between four independent variables (enzyme addition, enzymolysis time, solid–liquid ratio, enzymolysis temperature,) and response value (SDF yield). The appropriate ranges of these independent variables were obtained based on single-factor experiments, and a four-factor and three-level RSM was established by Box–Behnken design (BBD). The specific factors and levels are shown in Table 5.

### 3.5. Purification of SDF from Akebia trifoliata Peel

The crude SDF obtained under the optimal extraction conditions was diluted with distilled water to a specific concentration. Subsequently, a predetermined quantity of AB-8 macroporous resin was used for adsorption at 30 °C and 100 g/min for 2 h [40]. After filtration, the filtrate was mixed with Sevag reagent (chloroform: n-butanol = 3:1 (*v*/*v*)) at a volume ratio of 2:1 to repeatedly eliminate protein until no protein layer remained [41]. Following the removal of the organic solvent using a rotary evaporator (Kewei Yongxing Instrument Co., Ltd., Beijing, China), dialysis (Shanghai Yuanye Biotechnology Co., Ltd., Shanghai, China) was performed in distilled water for 72 h, with the water being replaced every 8 h. The purified SDF was finally lyophilized (AP-SDF) for subsequent analysis.

### 3.6. Characterization Analysis

#### 3.6.1. Chemical Composition Analysis

The protein content of AP-SDF was determined using the Bradford method [42], the total carbohydrate content was evaluated according to phenol–sulfuric acid method [43] and the uronic acid content was determined through the carbazole–sulfuric acid method [44].

#### 3.6.2. Monosaccharide Composition Analysis

The monosaccharide composition of AP-SDF was determined by ion chromatography [45]. Briefly, AP-SDF was hydrolyzed with 1 mL of 2 M trifluoroacetic acid (TFA) in a chromatographic bottle at 121 °C for 2 h. The sample was dried with nitrogen, washed with 99.99% methanol and dried again under nitrogen (three times), and then dissolved in sterile water for testing. The Dionex™ CarboPac™ PA20 (150 × 3.0 mm, 10 μm) liquid chromatography column was used in the ion chromatography system (ICS5000, Thermo Fisher Scientific, Waltham, MA, USA). The sample size was 5 μL, and the mobile phases were A (H_2_O), B (0.1 M NaOH) and C (0.1 M NaOH, 0.2 M NaAc) at a flow rate of 0.5 mL/min. The column temperature was 30 °C and the elution gradient was 0–60 min. Fucose, rhamnose, arabinose, galactose, glucose, xylose, mannose, fructose, ribose, galacturonic acid, glucuronic acid, mannose acid and gururonic acid were used as monosaccharide standards.

#### 3.6.3. Molecular Weight (Mw) Analysis

The molecular weight (Mw) of AP-SDF was determined by gel chromatography- refractive index–multi-angle laser light scattering system [46]. The liquid phase system was a U3000 (Thermo Fisher Scientific, Waltham, MA, USA), the differential refractive index detector was an Optilab T-rEX (Wyatt technology, Santa Barbara, CA, USA), and the laser light scattering detector was a DAWN HELEOS Ⅱ (Wyatt technology, Santa Barbara, CA, USA). Gel exclusion columns (Ohpak SB-805 HQ (300 × 8 mm), Ohpak SB-804 HQ (300 × 8 mm) and Ohpak SB-803 HQ (300 × 8 mm)) were used in series. The AP-SDF with a concentration of 1 mg/mL was prepared by mobile phase (0.02% NaN_3_, 0.1 M NaNO_3_), and the sample size was 100 μL through a 0.45 μm filter. The column temperature was 45 °C and the flow rate was 0.5mL/min. The Mw was calculated by comparing the calibration curves of glucose and dextran standard.

#### 3.6.4. Fourier Transform–Infrared (FT-IR) Spectroscopy Analysis

The structure and functional groups of AP-SDF were analyzed by FT-IR, based on the method of Vandanjon et al. [47]. Briefly, AP-SDF was mixed with 200 mg KBr and pressed into 1mm sheets. Then, an FT-IR spectrometer (Nicolet iZ-10, Thermo Fisher Scientific, Waltham, MA, USA) was used for analysis in the scanning range of 4000–400 cm^−1^ with 32 scans.

#### 3.6.5. X-ray Diffraction (XRD) Analysis

The crystal structure of AP-SDF was analyzed by X ‘Pert Pro X-ray diffractometer (PANalytical, Almero, Haute-ethel, Netherlands). The diffraction conditions were: a copper target Cu-Kα was used with a voltage of 40 kv and a current of 30 mA, a scanning range of 5–60°, a scanning step of 0.02° and a scanning speed of 4°/min [48].

#### 3.6.6. Scanning Electron Microscope (SEM) Analysis

A scanning electron microscope (HITACHI Regulus 8100, Hitachi, Tokyo, Japan) was used to observe the shape and microstructure of AP-SDF [49]. The sample was applied to the conductive carbon tape, sputtered with a layer of gold, and the scanning electron microscope images were recorded at an accelerating voltage of 15 kV and images were taken under different magnifications.

#### 3.6.7. Ultraviolet (UV)–Visible Spectroscopy Analysis

The full-band ultraviolet spectrum of AP-SDF solution (5 mg/mL) was scanned with a microplate reader (Multiskan GO, Thermo Fisher Scientific, Waltham, MA, USA) at 200–1000 nm. 

### 3.7. Functional Properties

#### 3.7.1. Water/Oil-Holding Capacity (WHC/OHC) Analysis

The WHC and OHC were determined based on the method by Si et al. [50], with minor modification. A 0.05 g sample of AP-SDF was accurately weighed in a 10 mL centrifuge tube, and 5 mL of distilled water or rapeseed oil was added. The mixture was thoroughly shaken and allowed to stand at 37 °C for 24 h. The mixtures were then centrifuged at 1776× *g* for 20 min. The supernatant was discarded and the residue was weighed. The WHC or OHC were calculated using the following formula:

(3) $$WHC\;and\;OHC\;(g/g)=\frac{M_{1}-M}{M}$$

where *M*_1_ is the weight of the sample after water or oil holding (g), and *M* is the dry weight of the sample (g).

#### 3.7.2. Swelling Capacity (SC) Analysis

The SC was determined according to the method reported by Xu et al. [21]. A 0.1 g sample of AP-SDF was accurately weighed into a 10 mL centrifuge tube, the initial volume was recorded and 5 mL of distilled water was added. After the sample was soaked and allowed to stand at room temperature for 24 h, the volume of AP-SDF after swelling was recorded. The SC was calculated as follows:

(4) $$SC\;(mL/g)=\frac{V_{1}-V}{M}$$

where *V*_1_ is the volume of the sample after swelling (mL), *V* is the initial volume of the sample (mL), and *M* is the dry weight of the sample (g).

### 3.8. Bioactivity Analysis

#### 3.8.1. Antioxidant Activity

The in vitro antioxidant capacity of AP-SDF in vitro was determined using DPPH, ABTS, hydroxyl and FRAP assays, as previously described [36,51], with minor modifications. AP-SDF was configured into different concentrations of solution for use. The absorbance of the reaction mixture was recorded at 517 nm (DPPH), 734 nm (ABTS), 510 nm (hydroxyl) and 593 nm (FRAP) using a microplate reader. The free radical scavenging rate was calculated using Formula (4). The FRAP value (mmoL FeSO_4_/g) was calculated according to the standard curve of FeSO4, and Vc was used as positive control.

(5) $$\mathrm{Scavenging}\;\mathrm{rate}\;(\%)=(\frac{A_{0}-\;(A-A_{1})}{A_{0}})\;\times 100$$

where *A*_0_ is the absorbance of the blank control, *A*_1_ is the absorbance of the sample background, and *A* is the absorbance of the sample.

#### 3.8.2. α-Glucosidase Inhibitory Activity

The α-glucosidase inhibitory activity was measured by the method previously described [52], with slight modifications. Briefly, a 0.33 U/mL concentration of α-glucosidase and 5.0 mM p-nitrophenyl-α-D-glucopyranoside (pNPG) were prepared with 0.1 M pH = 6.8 phosphate buffer solution (PBS). A 50 μL sample of AP-SDF solution at a certain concentration was mixed with 100 μL of α-glucosidase in 96-well microtiter plates. After a 10 min reaction at 37 °C, 50 μL of pNPG was added and then incubated at 37 °C for 20 min. The reaction was terminated by adding 100 μL of 0.2 M Na_2_CO_3_ solution, and the absorbance was measured at 405 nm. Acarbose was used as a positive control. The α-glucosidase inhibition rate was calculated according to the following formula:

(6) $$Inhibition\;rate\;(\%)=\frac{A_{0}-\;(A-A_{1})}{A_{0}}\times 100$$

where *A*_0_ is the absorbance of PBS instead of sample, *A*_1_ is the absorbance of PBS instead of α-glucosidase and pNPG, and *A* is the absorbance of the sample.

#### 3.8.3. Cholate Binding Activity

The cholate binding activity of AP-SDF was measured according to the method of Wang et al. [53], with slight modifications. A 0.5 mL sample of AP-SDF solution at different mass concentrations was placed in a centrifuge tube, and 0.5 mL of HCl (0.01 M) solution and 1.5 mL of pepsin (10 mg/mL) were added. The mixture was shaken at 37 °C for 1 h to simulate gastric digestion. After adjusting to pH 6.3, 2 mL of trypsin (10 mg/mL) was added and the mixture was shaken at 37 °C for 1 h to simulate intestinal digestion. Then, 2 mL of cholate solution (0.3 mM) was added and the mixture was shaken at 37 °C for 20 min, centrifuged at 4000× *g* for 20 min, and 0.25 mL of supernatant was taken. After adding 0.75 mL of 60% H_2_SO_4_, the mixture was heated at 70 °C for 25 min, then cooled in an ice bath for 5 min. A 200 μL sample was added to a 96-well microtiter plate, and the absorbance was measured at 387 nm. The cholate content was determined based on the standard curves and the cholate binding rate was calculated as follows:

(7) $$Binding\;rate\;(\%)=\frac{C-C_{1}}{C}\times 100$$

where *C* is the amount of cholate before adsorption (mmol/L), and *C*_1_ is the amount of cholate after adsorption (mmol/L).

#### 3.8.4. Cholesterol Adsorption Capacity (CAC)

The CAC of AP-SDF was evaluated based on a previous method [54], with slight modifications. Egg yolk was whipped with 9 volumes of distilled water and fully stirred into an emulsion. The emulsion (15 mL) was mixed with AP-SDF (0.05 g) at pH 7.0 or 2.0, respectively, and then the mixtures were oscillated at 37 °C for 2 h and centrifuged at 1776× *g* for 20 min. A 0.04 mL sample of supernatant was absorbed in the test tube and diluted with acetic acid (1:10 *v*/*v*). The cholesterol content was determined according to the standard curve preparation method of the o-phthalaldehyde method, and the CAC was calculated as follows:

(8) $$CAC\;(mg/g)=\frac{M_{0}-M_{1}}{M}$$

where *M*_0_ is the content of cholesterol in the emulsion (mg), *M*_1_ is the content of cholesterol after adsorption (mg), and *M* is the weight of AP-SDF (g).

### 3.9. Statistical Analysis

The results were expressed as mean ± standard deviation (*n* ≥ 3). One-way analysis of variance (ANOVA) and Duncan’s multiple range tests in SPSS 25.0 software (IBM, Chicago, IL, USA) were used to analyze the significant differences (*p* < 0.05). The response surface optimization was performed using Design-Expert v10 software (Stat-Ease, Minneapolis, Mn, USA). GraphPad Prism 9.5.1 (GraphPad Software, San Diego, CA, USA) and Excel 2019 software were used for data analysis and graph processing.

## 4. Conclusions

Our study demonstrates that the yield of SDF from *Akebia trifoliata* peel can be significantly enhanced by optimizing the complex enzymatic extraction process. The optimized yield could reach up to 20.87%. The purified AP-SDF, an acidic polysaccharide primarily composed of arabinose, galacturonic acid, and glucose, exhibited an Mw of 95.52 kDa and contained a certain amount of uronic acid. Structurally, AP-SDF displayed characteristic polysaccharide peaks and a porous honeycomb structure, aligning it with the cellulosic type I polysaccharide found in most plant polysaccharides. Functionally, AP-SDF demonstrated notable water-holding, swelling, antioxidant, hypoglycemic and hypolipidemic capacities. Notably, the oil-holding capacity and cholesterol adsorption capacity of AP-SDF surpassed those reported in previous studies, likely due to its unique structural characteristics. These findings contribute to the development of functional products with antioxidant, hypoglycemic and hypolipidemic activities, thereby promoting the high-value utilization of *Akebia trifoliata* waste. As this study is foundational research on AP-SDF, its biological activities are limited to in vitro observations. Therefore, further research is warranted to validate these biological activities, elucidate the specific mechanisms of action, and establish the structure–activity relationship through animal experiments.

## Figures and Tables

**Table 1:** Box–Behnken design and results.

Run	* X *_ 1 _ Enzyme Addition (U/g)	* X *_ 2 _ Enzymolysis Time (min)	* X *_ 3 _ Solid–liquid Ratio (g/mL)	* X *_ 4 _ Enzymolysis Temperature (°C)	* Y * SDF Yield (%)
1	−1	−1	0	0	17.04
2	1	−1	0	0	16.49
3	−1	1	0	0	16.07
4	1	1	0	0	16.93
5	0	0	−1	−1	18.45
6	0	0	1	−1	16.01
7	0	0	−1	1	18.92
8	0	0	1	1	17.85
9	−1	0	0	−1	14.31
10	1	0	0	−1	14.92
11	−1	0	0	1	15.68
12	1	0	0	1	16.15
13	0	−1	−1	0	19.74
14	0	1	−1	0	19.87
15	0	−1	1	0	18.63
16	0	1	1	0	18.98
17	−1	0	−1	0	16.14
18	1	0	−1	0	16.77
19	−1	0	1	0	14.81
20	1	0	1	0	15.21
21	0	−1	0	−1	18.26
22	0	1	0	−1	18.22
23	0	−1	0	1	19.05
24	0	1	0	1	19.13
25	0	0	0	0	20.64
26	0	0	0	0	20.82
27	0	0	0	0	20.54
28	0	0	0	0	20.19
29	0	0	0	0	20.81

**Table 2:** ANOVA of regression model.

Source	Sum of Squares	Df	Mean Square	*F *-Value	*p*-Value	Significance
Model	110.48	14	7.89	121.05	<0.0001	**
*X*_1_	0.49	1	0.49	7.49	0.0161	*
*X*_2_	8.33 × 10^−6^	1	8.33 × 10^−6^	1.2 8 × 10^−4^	0.9911	
*X*_3_	5.88	1	5.88	90.19	<0.0001	**
*X*_4_	3.64	1	3.64	55.85	<0.0001	**
*X*_1_ *X*_2_	0.50	1	0.50	7.62	0.0153	*
*X*_1_ *X*_3_	0.013	1	0.013	0.20	0.6593	
*X*_1_ *X*_4_	4.90 × 10^−3^	1	4.90 × 10^−3^	0.075	0.7880	
*X*_2_ *X*_3_	0.012	1	0.012	0.19	0.6732	
*X*_2_ *X*_4_	3.60 × 10^−3^	1	3.60 × 10^−3^	0.055	0.8176	
*X*_3_ *X*_4_	0.47	1	0.47	7.20	0.0178	*
*X*_1_^2^	89.74	1	89.74	1376.56	<0.0001	**
*X*_2_^2^	0.35	1	0.35	5.42	0.0355	*
*X*_3_^2^	8.02	1	8.02	123.05	<0.0001	**
*X*_4_^2^	18.00	1	18.00	276.10	<0.0001	**
Residual	0.91	14	0.065			
Lack of Fit	0.65	10	0.065	0.97	0.5607	not significant
Pure Error	0.27	4	0.066			
Cor Total	111.39	28				
R^2^ = 0.9918		R^2^_Adj_ = 0.9836		Adeq Precision = 33.616		C.V. % = 1.43

Note: ** *p* < 0.01, extremely significant difference; * *p* < 0.05, significant difference.

**Table 3:** Chemical composition, monosaccharide composition and molecular weight distribution of AP-SDF.

Characteristics	Values
Chemical Composition (%)	
Total Carbohydrate Content	52.46 ± 0.13
Protein Content	8.33 ± 0.44
Uronic Acid Content	34.90 ± 0.31
Monosaccharide Composition (%)	
Fucose	0.50 ± 0.01
Arabinose	36.03 ± 0.36
Rhamnose	7.16 ± 0.47
Galactose	5.43 ± 0.16
Glucose	19.00 ± 0.24
Xylose	2.79 ± 0.02
Mannose	1.25 ± 0.02
Galacturonic Acid	27.40 ± 1.01
Glucuronic Acid	0.44 ± 0.05
Mw(kDa)	
Mw	95.52
Mn	37.13
Mp	55.63
Mz	645.82
Mw/Mn	2.57

Note: Values are presented as means ± standard deviations (*n* = 3).

**Table 4:** WHC, OHC and SC of AP-SDF.

Sample	WHC (*g*/*g*)	OHC (*g*/*g*)	SC (mL/g)
AP-SDF	1.31 ± 0.16	15.11 ± 0.60	4.68 ± 0.47

**Table 5:** Factors and levels of RSM.

Factors	Levels		
	−1	0	1
*X*_1_ Enzyme Addition (U/g)	450	600	750
*X*_2_ Enzymolysis Time (min)	80	100	120
*X*_3_ Solid–Liquid Ratio (g/mL)	1:20	1:25	1:30
*X*_4_ Enzymolysis Temperature (°C)	45	50	55

## Data Availability

Data are contained within the article.
